# Architecture for Improving Terrestrial Logistics Based on the Web of Things

**DOI:** 10.3390/s120506538

**Published:** 2012-05-18

**Authors:** Miguel Castro, Antonio J. Jara, Antonio Skarmeta

**Affiliations:** Clinical Technology Lab, Computer Science Faculty, University of Murcia, Murcia, 30003, Spain; E-Mails: jara@um.es (A.J.J.); skarmeta@um.es (A.S.)

**Keywords:** internet of things, web of things, RFID, logistics, intelligent transportation systems, tracking, traceability, monitoring

## Abstract

Technological advances for improving supply chain efficiency present three key challenges for managing goods: tracking, tracing and monitoring (TTM), in order to satisfy the requirements for products such as perishable goods where the European Legislations requires them to ship within a prescribed temperature range to ensure freshness and suitability for consumption. The proposed system integrates RFID for tracking and tracing through a distributed architecture developed for heavy goods vehicles, and the sensors embedded in the SunSPOT platform for monitoring the goods transported based on the concept of the Internet of Things. This paper presents how the Internet of Things is integrated for improving terrestrial logistics offering a comprehensive and flexible architecture, with high scalability, according to the specific needs for reaching an item-level continuous monitoring solution. The major contribution from this work is the optimization of the Embedded Web Services based on RESTful (Web of Things) for the access to TTM services at any time during the transportation of goods. Specifically, it has been extended the monitoring patterns such as observe and blockwise transfer for the requirements from the continuous conditional monitoring, and for the transfer of full inventories and partial ones based on conditional queries. In definitive, this work presents an evolution of the previous TTM solutions, which were limited to trailer identification and environment monitoring, to a solution which is able to provide an exhaustive item-level monitoring, required for several use cases. This exhaustive monitoring has required new communication capabilities through the Web of Things, which has been optimized with the use and improvement of a set of communications patterns.

## 1. Introduction

At present, the continuous development of Information Technology and Communications offers a range of possibilities large and versatile enough to meet the needs of contemporary society in the creation, management, maintenance and dissemination of information. However, new needs appear constantly, for which we must provide solutions that offer to its potential users, a number of services that meet, as far as possible, those needs, while anticipating new requirements that may arise in the future.

In the transportation of goods, there are many products and services offered today, but there is a non-stop appearance of new ideas and solutions through the use of Information Technology and Communications, to facilitate and improve productivity in the work related to the various processes that are encompassed within this sector. So much so, that research in the field of Intelligent Transportation Systems (ITS) has long been one of the booming industries in the sector of informatics and communications, and where a constant investment in its development exists, carried out by numerous companies, government agencies and even states themselves.

In recent years, many of these solutions have been integrated into all types of freight vehicles [1]. In this regard, one of the more popular services among the companies involved in logistics has been the implementation at various levels, of several approaches for fleet management. These systems, based on the use of global positioning technologies and communications, such as Global Positioning System (GPS) and Global System for Mobile Communications/General Packet Radio Service (GSM/GPRS), respectively, are able to indicate the position of each vehicle that a particular fleet has and provide some additional information regarding various aspects relating to vehicles in which communications systems have been installed.

However, the current trend leads to systems which go one step further, allowing the tracking of vehicles, the implementation of intelligent control on the charge status and identifying the products being transported at one point.

In this paper, a proposal based on sensors that integrate next generation connectivity for smart objects or smart things (6LoWPAN, IPv6 Low power Wireless Personal Area Network) and radio frequency identification technology (Radio Frequency Identification—RFID) will be presented. Specifically, the solution lays in using Sun Small Programmable Object Technology (SunSPOT), developed by Sun Labs, currently owned by Oracle, which conveniently has been extended to support RFID integration and access to information through a set of services based on Representational State Transfer (REST), and presents a novel system based on the Internet of Things (IoT) that enables monitoring, tracking, tracing and control of road transportation, with the aim of obtaining high-level management, helping to fulfill the respective legislation on traceability of products, and ensure safe transportation process and being capable of ensuring the good condition and quality of shipped products. However, the use of nomadic devices in the interior of cars and trucks supposes a scalability problem as the number of services grows [2].

Several commercial products and *ad hoc* solutions have been developed for improving fleet management in logistics companies. As a next step in this frame, the ICT application in the supply chain is extending towards better control not only of vehicles, but also of products. The research world in ITS, electronics and food technologies has worked during the past years on this issue, even overcoming tracking approaches to monitor the product status by means of sensor systems.

Technological advances for improving efficiency should attend to three key challenges for managing goods [3]: tracking, tracing and monitoring (TTM). The most extended one, tracking, focuses on the ability to locate vehicles and freight at any time. Tracing allows logistics companies and even final consumers to know movements of goods from the source to the final destination point. Finally, the most recent challenge is continuous monitoring, which enables logistics companies to assure product quality during transportation. Among the technologies that are being applied in TTM solutions, new short-range wireless communications require special attention. RFID technologies play a key role in the detection of products, by means of a tagging scheme where a reader identifies products at a distance of meters.

The platform presented in this project proposes a comprehensive solution to the problems discussed in the articles above by installing an arch with multiple RFID readers at the loading area of the truck, as proposed in [4]. In this case, pallets, or boxes containing products are marked with RFID tags that integrate technology and incorporate an Electronic Product Code (EPC) associated unambiguously to identify any object to which they are attached, thus an item can be detected when it enters or leaves the loading area of the truck when passed through the arch, allowing comprehensive management of goods. However, the developments described above for these systems, lead not only to control the traceability of products, but also to monitor the load and the surrounding environment. Using the experimental SunSPOT technology, this project examines to meet the needs of monitoring and identifying perishable goods and foodstuffs during transport.

Vaccines, drugs and clinical trials products need to be shipped within a prescribed temperature range to maintain their efficacy as well as food to maintain its freshness. Active RFID allows having a complete history of the temperature exposure of the perishables thus allowing predicting the remaining shelf-life [5]. The pharmaceutical and food industries can use this data to calculate when to replenish perishables and how much product to put on shelves. The benefits of applying RFID and sensors to perishable goods include improved food and drugs safety, longer vaccines and drug efficacy, more efficient product recalls, reduced costs due to less spoilage, lower inventories, more efficient logistics, and improved customer service.

The proposed system integrates secure identification and offers RFID tracking and tracing through a distributed architecture for heavy goods vehicles, while the environment in which the goods are transported is continuously monitored (temperature, humidity and lighting) through the use of active sensors embedded in the SunSPOT nodes, benefiting from the flexibility they offer when it comes to be deployed and configured wirelessly. In addition to the sensors integrated directly into the plate external sensors are used to extend the functionality and versatility of the system. In this regard, it is possible to install new sensors through, on the one hand, digital and analog inputs and outputs, and on the other hand, the serial communication port (USART) integrated in SunSPOT. It is intended, therefore, to develop a tracking, monitoring and traceability system for the transport of goods that meets the following objectives:

1. Develop a comprehensive system that offers a flexible architecture and easy integration, while being scalable according to specific needs.
2. The architecture will be composed of entities that maintain a high level of homogeneity, in order to facilitate the tasks of development, system configuration and deployment in environments with high heterogeneity.
3. Access to services for monitoring, tracking and traceability may be required during the course of goods transportation. To this end, it must not be limited to offering these services only at specified times (loading and unloading of products, beginning or end of route, *etc.*), but services should be accessible en-route. To this end, a series of developed Web Services through the integration of REST technology will support this functionality.
4. Moreover, in addition to meeting the needs of monitoring, tracking and traceability described above, there is the need to provide appropriate services to carry out an exhaustive item-level monitoring for cases in which the importance of maintaining a certain product within a temperature range, humidity and/or lighting conditions is an important factor.
5. It will be necessary to consider various aspects of security related to identification of products through wireless identification technology, as well as in regard to external communications.
6. Different ways for resource access will be provided, in order to make the system as flexible and versatile as possible. This will offer end users the opportunity to set their own mash-ups for access to information, depending on the role they play in gaining access to it, being also possible to easily configure remote control server applications.

## 2. Related Works

Beyond the simple management of vehicles, which comprise a fleet, at present, certain ways of work are essential, related not only to the management of the vehicle itself, but going a step further, related to goods carried within them. Therefore a new set of technologies is necessary to meet the needs that allow us to be able to offer services that enable monitoring, tracking and traceability of products contained inside the cargo area of vehicles that come into play to implement this new solution.

For the tracking tasks described above, GPS technology has already been well established, by which it is possible to determine the absolute position of a particular vehicle, whose position can be associated with an existing map, obtaining additional information about the path that the vehicle on which the receiver is installed.

With regard to the tasks of traceability and monitoring, there are a variety of open fronts in order to find optimal ways for both the development of a traceability system, which can trace the road made for a certain product during transport at all times and for the implementation of a monitoring system which, through sensors within the cargo areas of vehicles, offers the possibility of knowing the state of environment in which a product is immersed at a precise moment. As mentioned above, one of the main objectives of the proposed solution is to reach a level of comprehensive item monitoring and tracking.

Especially relevant is the intention expressed by the European Union to regulate the transport of goods such as pharmaceuticals, food, animals and dangerous goods [6]. To this end, it is necessary to implement a comprehensive system of traceability in the food and pharmaceutical businesses, together with the services provided by transport companies, able to carry out specific and precise withdrawal of products, or to inform consumers or control officials and avoid further unnecessary disruption in the case of food safety problems and/or care, thus ensuring that in a situation like this, it will be possible to identify the source that supplied the drugs, foods, animal feed or substances that can be incorporated in a food or feed, and to ensure traceability at all stages allowing for an investigation. Furthermore, the support from the European Union is clear, with financing of several actions in the area of ITS such as the ITSSv6 project. The objective of the ITSSv6 project is to develop a reference open-source IPv6 ITS Station stack freely available to European and national third parties (projects, industry and academia) using IPv6 for Internet-based communications in Field Operational Tests (FOTs) of Cooperative Systems. The project aims at further specifying the concept of the ITS Station as standardized by ISO TC204 WG16 (CALM) and ETS TC ITS with brushed up existing and additional IPv6 features required for operational deployment of Cooperative Systems, *i.e.*, enhanced performance, embedded security, remote management of deployed systems and ease of configuration. The project takes as an input the FP6 CVIS core communication software and additional modules developed by FP7 GeoNet and produce an enhanced IPv6 ITS Station stack adapted to operational use in large scale Field Operational Tests. From a specification viewpoint, the IPv6 features are well integrated within the overall ITS Station architecture (particularly ITS Station management and ITS facilities). As a result, the IPv6 software release fits nicely with recommended physical interfaces (802.11p and 3G) and benefit to Cooperative Systems applications (road safety, traffic efficiency and infotainment types of applications which require Internet communications).

Coming back to the technologies involved in ITS solutions, it is clear that the development of different navigation and communication techniques has greatly enriched the solutions for the monitoring of ground transportation and fleet management. In this sense, many works deal with proposals for integrating various technologies in freight systems, and to improving the efficiency of supply chains. Among them, the radio frequency identification technology and wireless sensors (obeying the guidelines of the IEEE protocol 802.15.4) occupy, at present, the areas of greatest interest in research on these issues. In [7] an indoor traceability system for direct application in stores is described. This case study is exploited in [8], and also incorporates a system of decision support for container management in a warehouse. However, with required tasks, either tracing or tracking, RFID by itself is unable to meet the economic requirements and effectiveness, because of the need to deploy a large and expensive infrastructure of readers. Referring to this, in [9] some major challenges to overcome in the global adoption of RFID technology are presented. Related to Wireless Sensor Networks, there are interesting works focused on the implementation of real-time data management protocols as exposed in [10]

The evolution of tracking and tracing systems has been directed towards monitoring the freight status [11]. Applying SunSPOT [12], this research examines how to meet the needs of monitoring, identification and control during the transportation process of perishable goods and food products. The system integrates secure RFID identification and traceability in a distributed architecture for trucks while maintaining monitoring of the environment in which those goods are involved (temperature and lighting) based on active sensors that every SunSPOT node implements, taking advantage from the benefits and versatility these motes offer.

In terms of the evolution of communication technologies, in conjunction with the development of new devices such as wireless personal devices, embedded systems and smart objects, mixed together with the innovation of the services with the definition of cloud computing, online services, and ubiquitous access to information, an extension of the capabilities of the current Internet is defined, that make feasible connecting to the Internet all the objects and devices which are found surrounding us, the so-called IoT [13], which is one of the Future Internet foundations. The objective of IoT allows systems to gain total control and access to other systems leading to the provision of ubiquitous communication and computing. Thereby a new generation of smart and small devices, context awareness services and applications can be defined.

The mentioned tremendous increase of the use of Internet, from some 360 million users in 2000 to 1.6 American billion Internet users and 4 American billion mobile users with over 570 million Internet-enabled hand-held devices is only the beginning, since it is estimated that the extension of the Internet to smart things, will reach by 2020 between 50 to 100 billion devices connected to the Internet [14]. This growth have led to the IPv4 address exhaustion at the beginning of 2011 prompting enterprises and academia to begin implementing, integrating and moving to IP Version 6 (IPv6), the next-generation Internet protocol which presents an increase of the Internet capabilities and addressing space. Related to that, some approaches related to this topic, taking advantage of these new capabilities are exposed in [15]. This grows of the Internet presents serious problems for scalability, manageability, addressing/identity, and robustness, and the openness and ubiquity features of the Internet presents problems to offer a suitable support for security, privacy, and secure mobility, especially in cases where the information involved in the process is extremely compromised, such as clinical environments [16]. Therefore a new redesign of the Internet architecture and definition of new security aspects are required to solve the mentioned problems for the Future Internet of Things [17]. For this purpose, several projects from industrial and international collaboration are being carried out to define the Future Internet architecture, which solves the limitations of the current architecture [18].

The Future Internet of Things presents the requirements of managing security and privacy, which present several changes, since on the one hand, the innovative services and solutions considered for the Future Internet require a global interoperability and mobility support, but on the other hand requires a separation between the global networks and from the edge networks, since they are highly constrained in terms of computational capabilities, memory, communication bandwidth, and battery power, which present vulnerabilities for the security and privacy problems addressed in the current Internet [19]. 6LoWPAN offers the Future Internet of Things all the advantages from the Internet Protocol (IPv6) such as scalability, flexibility, ubiquity, openness, and end-to-end connectivity, and it could be also considered ideally that 6LoWPAN devices are powered with the current Internet protocols for management, e.g., Simple Network Management Protocol (SNMP), and security, e.g., IP Security (IPSec). However it is not feasible for the 6LoWPAN devices to be associated with host-based protocols because of the mentioned constrains.

Therefore, with the mentioned constrains and requirements from the Internet of Things for security, privacy, mobility and scalability, an architecture which addresses and solves all these issues is required, incorporating a specific design for solutions and protocols.

In the following sections the solution adopted is exposed, taking into account the needs and considerations determined after a process of analysis of the studies mentioned above, with particular emphasis on what have been the strategies and/or technologies chosen to satisfy the requirements and main objectives of the described problem. In Section 3, the overall architecture is exposed, giving a general explanation of the solution developed, and in the next subsections how the different devices and sensors used have been integrated in order to carry out the work is explained. Section 4 describes the different technologies and approaches considered in order to fulfill all the information access and treatment requirements. Section 5 introduces a specific evaluation for the proposed architecture, in order to show how it meets the requirements and how it is possibly to manage it in a clearly and transparently manner and finally, in Section 6, final conclusions are given.

## 3. Overall Architecture

**Figure 1.**
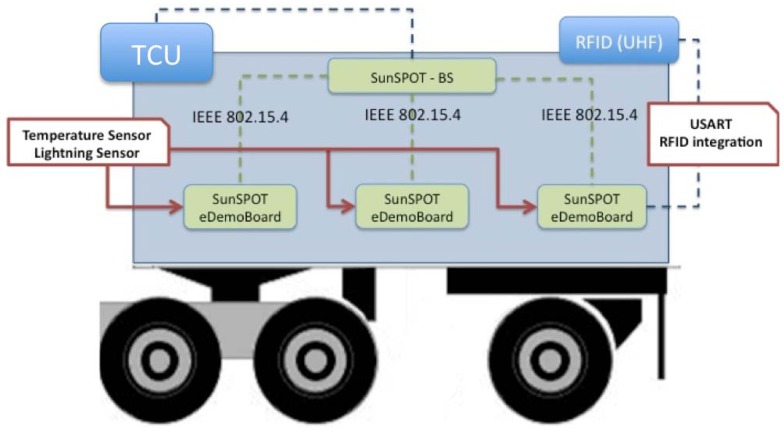
Tracking and Monitoring Architecture of the System installed inside the truck.

The proposed tracking and monitoring architecture, which entirely consists in SunSPOT nodes, and was developed under this project, is presented in Figure 1. It comprises the Trailer Control Unit (TCU), which shows the great homogeneity level achieved by the use of this technology in which lighting and temperature sensors, in addition to the RFID reader rack connected to the node located at the trailer loading door. Different sensors are integrated directly in each monitoring entity compared to other proposed architectures where each sensor could implement a different technology, leading to heterogeneous systems, making deployment and configuration processes more difficult.

### 3.1. Tracking and Monitoring Architecture

The deployed nodes offer IPv6 over IEEE 812.15.4 connectivity allowing direct access through any device with Internet connectivity. Additionally, integrating RFID technology into our system gives us the ability to uniquely identify the products that enter, exit, and that are found inside the truck at any point by reading the Electronic Product Code (EPC) present in the RFID tags attached to them. Thus, the system is able to provide tracking and monitoring services in an efficient manner. As previously mentioned, the architecture also has a dedicated monitoring system, based on active tags tracking the item-level status of the goods with its corresponding UHF dedicated readers for making this task feasible.

In order to make it even more accessible the data recorded by the system, in agreement with the philosophy of Internet of Things, can be accessed through Web Services technology that integrates REST, *i.e.*, the previously mentioned Web of Things Architecture.

REST-style architectures consist of clients and servers. Clients initiate requests to servers; servers process requests and return appropriate responses. Requests and responses are built around the transfer of representations of resources. A resource can be essentially any coherent and meaningful concept that may be addressed. A representation of a resource is typically a document that captures the current or intended state of a resource.

At any particular time, a client can either be in transition between application states or “at rest”. A client in a rest state is able to interact with its user, but creates no load and consumes no per-client storage on the servers or on the network. The client begins sending requests when it is ready to make the transition to a new state. While one or more requests are outstanding, the client is considered to be in transition. The representation of each application state contains links that may be used next time when the client chooses to initiate a new state transition.

REST was initially described in the context of HTTP, but it is not limited to that protocol. RESTful architectures can be based on other Application Layer protocols if they already provide a rich and uniform vocabulary for applications based on the transfer of meaningful representational state. RESTful applications maximize the use of the pre-existing, well-defined interface and other built-in capabilities provided by the chosen network protocol, and minimize the addition of new application-specific features on top of them.

The application of this technology manages to offer client-server architecture in which web services are seen as resources and can be identified by their URIs (Uniform Resource Identifiers). Different operators wishing to access these resources can create the data represented the way most convenient to them, accessing them through a series of defined methods that are responsible for providing the required information, while offering the possibility of remotely modifying these data in the event of a necessary operation. Thus we synthesize information so that it can be automatically processed by applications that make use of the offered methods.

External communications will be through the integrated on-board unit in the truck OBU. This module will be responsible for acting as a gateway between the network infrastructure deployed inside the truck and the external data network via GPRS communication module.

### 3.2. SunSPOT Platform

**Figure 2.**
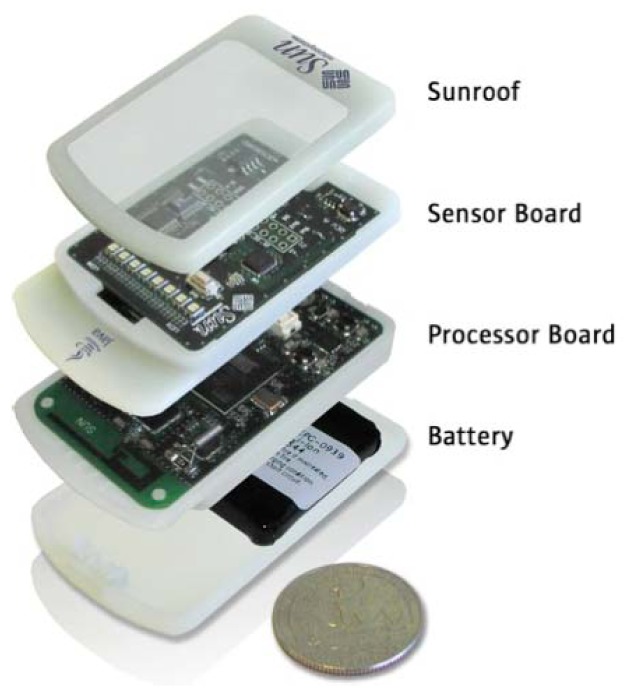
SunSPOT eSPOT device.

SunSPOT is an embedded platform with the aim of optimizing Java technology applications over Wireless Sensor Networks based on IEEE 802.15.4 (Figure 2). This present several advantages with respect to the previous nodes and devices found in the market for the development of sensor networks, such as high performance microprocessors based on ARM from Atmel, instead of the usual constrained solutions defined previously in the market, and mainly its extension of the current local communication domain to an IPv6-based global domain.

Finally, this offers an Operating System with support for Embedded Web Services. Those features are the reasons that make this platform able to reach a real Internet of Things. In addition, other features and advantages that are present in SunSPOT platform are having an extremely flexible hardware and software package, Java top to bottom, so the programming of new services and applications is such as easy as programming in conventional Java, moreover, even device drivers are written in Java. In addition to offering support for overlay networking such IPv6 over LoWPAN it also offers support for Mesh Networking and Multi-hop Over the Air Programming.

The current configuration of the Sun SPOT platform, the eSPOT, has a main processor running the Java VM Squawk and which serves as an IEEE 802.15.4 wireless network node. The SunSPOT is designed to be a flexible development platform, capable of hosting widely differing application modules.

**Figure 3.**
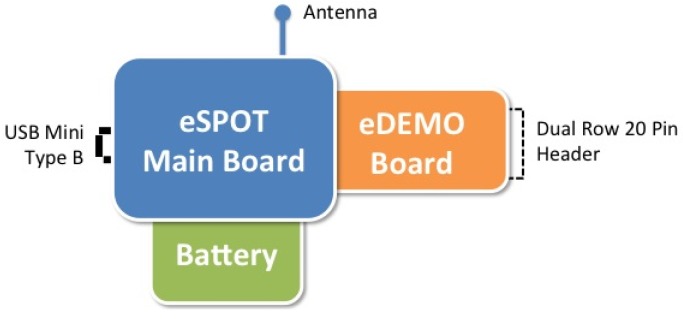
Block view of a SunSPOT eMainboard with an eDEMO board.

The selected configuration consists of a base station node, and three eSPOT nodes. Base station node has an eSPOT main board without a battery or an application board. In this case, power is supplied by a USB connection to a host workstation. The base station serves as radio gateway between other Sun SPOTs (and any other IEEE 802.15.4 devices) and the host workstation. eSPOT nodes contains the main board with a rechargeable LI-ION prismatic battery and an eSPOT daughterboard, the eDEMO board. Figure 3 shows the SunSPOT eMainboard general schema with an eDEMO board implemented on it.

From the point of view of hardware and sensors, the SunSPOT is composed of the denominated eDemo board that contains a 3-axis accelerometer, an ambient light sensor, a temperature sensor, eight tricolor LEDs, two push buttons, six analog input pads, four high-current high-voltage output pads, and five general I/O pads.

The SunSPOT eSPOT Main Board integrates an Atmel AT91RM9200 System on a Chip (SOC) integrated circuit. This unit incorporates the ARM920T ARM Thumb processor, based on the v4T ARM architecture ARM9TDMI. Power to the SOC is 3.0 V I/O voltage and 1.8 V core voltage. In normal operation, it consumes approximately 44 mW core power. The ARM executes at 180 MHz maximum clock speed based on a software controllable phase lock loop/oscillator. The SOC contains a 64-way associative 16 KB data cache and a 16 KB instruction cache. ARM9 uses standard ARMv4 memory management unit with 64-entry instruction TLB and 64-entry data TLB.

The SOC has a large collection of peripheral interface units. These include USB host port, USB device port, Ethernet MAC, programmable I/O (PIO) controller, serial peripheral interface (SPI) controller, TWI two-wire (I2C) interface, universal synchronous/asynchronous serial interface (USART), serial synchronous controller (I2S), multimedia card interface, three 16-bit counter/timers and systems timers. The unit also contains a real-time clock, which is unused. The SOC also contains a peripheral DMA controller (PDC) for fast direct access to USART, I2S, SPI, and memory channel.

**Figure 4.**
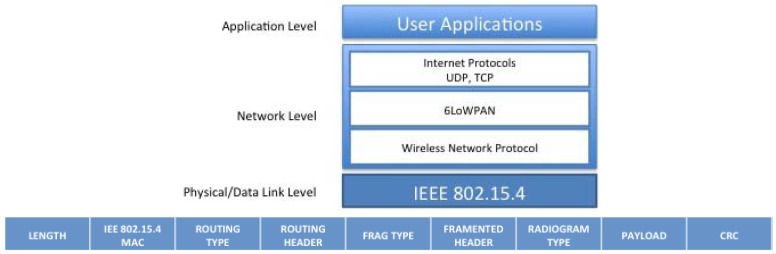
Layers structure and example of a radiogram packet.

From the network layer and radio communications point of view, SunSPOT offers a platform based on IEEE 802.15.4 as mentioned before. The wireless network communications uses an integrated radio transceiver, the TI CC2420 (ChipCon). The CC2420 is IEEE.802.15.4 compliant and operates in the 2.4 GHz to 2.4835 GHz ISM unlicensed bands. This presents a frame size of 127 bytes, which includes the considered headers from MAC layer, UDP, IP (these two last based on 6LowPAN). In Figure 4 is shown the structure of a conventional radiogram packet.

### 3.3. RFID Integration

**Figure 5.**
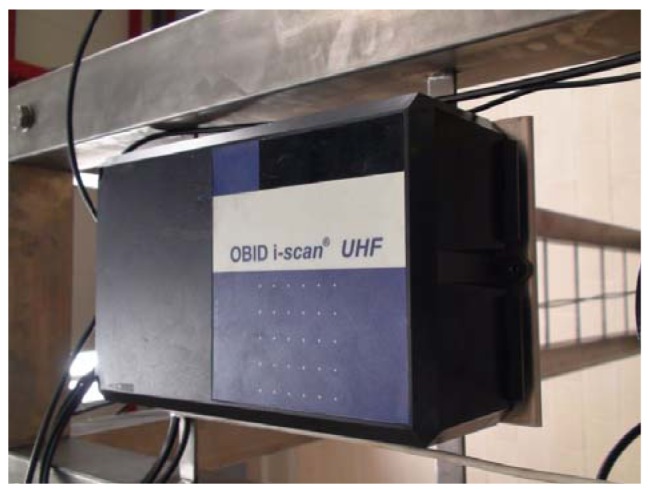
OBID i-scan UHF transponder for identification.

**Figure 6.**
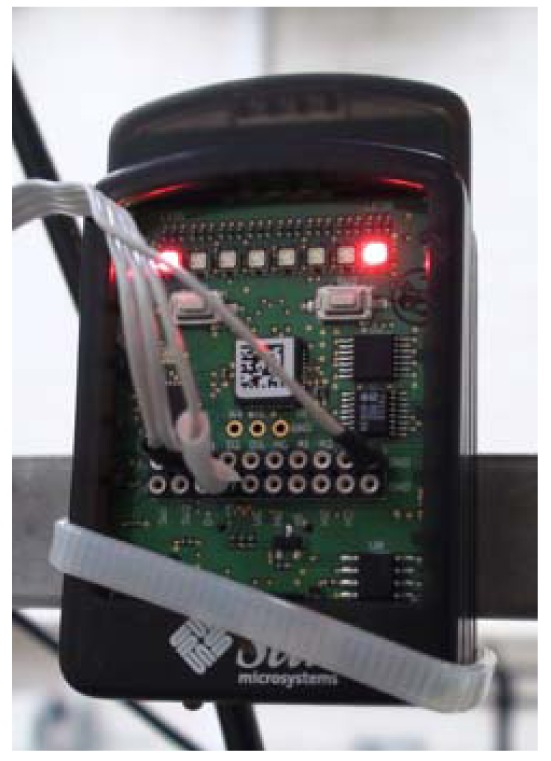
Detail of the serial RS232–USART interface.

As described above, one of the motes implements RFID reader connection through the SunSPOT USART, managing the tag reading functionality. The RFID reader selected for our development is an OBID i-scan UHF transponder for identification, working on the 865–960 MHz band, compatible with EPG Global and ISO/IEC 18000-6 (Figure 5) that can be connected to up to four external antennas and accessed through a serial RS232 interface (Figure 6).

**Figure 7.**
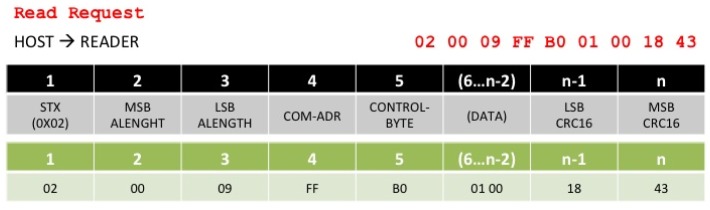
SunSPOT RFID communication protocol example.

**Figure 8.**
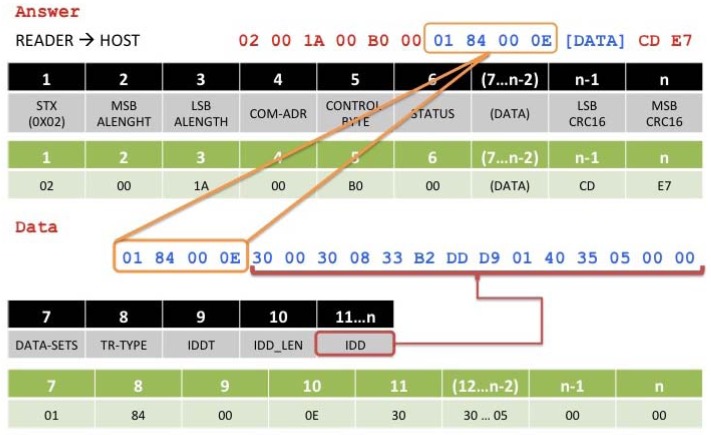
SunSPOT RFID communication protocol field correspondence.

The RFID installation in the trailer is based on a rack of antennas installed around the loading door thus improving the performance when detecting the passing of goods. One of the advantages of using SunSPOT nodes in our architecture is that they reduce the complexity of the solution deployment. When a new tag is discovered to be entering the truck, it is read and registered by the OBID i-scan, the read information is send via USART connection to the SunSPOT RFID adapted node whose communications protocol is based on the message format shown in Figures 7 and 8, where a processed request example is given.

When the scanner reads a new tag, all the data information related to this tag is extracted, in order to register it in the system for later access. In this way, data is presented as shown in Figure 8, where the different fields of interest when retrieving information from one tag are presented.

When extracting the information, notice that in addition to the tag information EPC code itself (IDD field), there are some other fields of interest related to the completed read such DATA-SETS, number of data sets read; TR-TYPE, type of transponder used (see Table 1); IDDT, data type identifier (see Table 2); IDD_LEN, identifier length, and finally the IDD itself.

Related to SunSPOT RFID integration, in Section 5 some other considered aspects in terms of information access and definition of application protocols for taking advantage of the Internet of Things approach capacities will be described.

### 3.4. Sensors Integration

The sensorised SunSPOT nodes used in our solution are responsible for regularly recording temperature and lighting values inside the truck. This task is done using the integrated temperature and lighting sensors of each sensorised node.

The Analog Devices ADT7411 ADC integrated in each SunSPOT node, converts analog inputs from the accelerometer, light sensor, and its own internal temperature sensor to digital values that can be read by SPOT applications. The ADC is also available to encode analog inputs from pins A0, A1, A2 and A3. In this way, some other sensors may be attached to the SunSPOT nodes in order to add new functionalities and measurement possibilities to the monitoring system in an easy manner.

Related to the temperature sensor, the ADT7411 ADC contains an internal temperature sensor that is capable of measuring temperatures in the range −40 to +125 degrees Celsius with an accuracy of 0.25 degrees Celsius. Because the temperature sensor is part of the ADT7411 chip, it actually measures the temperature on the chip, which is generally slightly different than the surrounding environment, especially during USB charging. The sensor gives the most accurate temperature readings immediately after the SunSPOT wakes up from deep sleep, when not connected to a USB.

The lighting sensor is mounted on top of the eDemo board. It is a Toshiba TPS851 light to voltage sensor. Output of the sensor is 0.1 V to 4.3 V, dark to light, using an emitter resistor of 4.7 K (R4). This voltage is buffered by op-amp U3 and divided by two resistor dividers R5 and R9. Output of the resistor divider is input to channel 5 of ADC U4 with an effective range of 0.05 V to 2.15 V. Peak sensitivity of the light sensor is 600 nm > 3dB sensitive is about ±45 and switching time is about 30 μsec.

#### 3.4.1. Externally Integrated Sensors

There has been integrated several external sensors through the USART communications port, concretely a light sensor and a temperature and humidity sensors have been used, both described in the following lines.

A TAOS TSL2550 light sensor has been integrated. This is a digital-output light sensor with a two-wire serial communication interface. It combines two photodiodes and an analog-to-digital converter (ADC) on a single CMOS integrated circuit to provide light measurements over an effective 12-bit dynamic range, with a response similar to that of the human eye.

Related to the temperature and humidity sensor, a Sensirion SHT11 single-chip multi-sensor module has been used for measurement of temperature and relative humidity. The device includes two calibrated micro-sensors for temperature and relative humidity, which are coupled to a 14-bit analogue-to-digital converter and a serial interface circuit. Conversion may be programmed with 8-, 12- or 14-bit accuracy, depending on application resolution or speed requirements. Humidity is measurable over 0–100% relative humidity, and temperature over the range −40 °C to 85 °C.

## 4. Remote Access and Client Services

**Figure 9.**
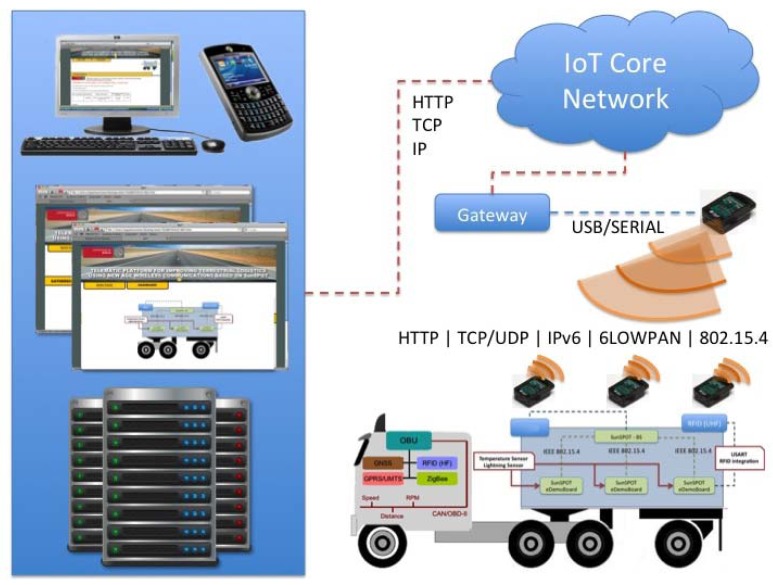
Schematic description of the different communication technologies used between entities.

Different services and applications have been developed to provide access to information in a manner best suited to the requirements to provide a particular service. In Figure 9, an explanatory chart describing the different technologies used to support these services and applications is shown. The following subsections describe the various technologies used in each part of the architecture and how, through the combination of these, the different services described can be provided.

### 4.1. Embedded Web Services

While the Internet protocol ties together heterogeneous networks into the Internet, the Web is a loosely coupled application layer architecture. Resources are key to the web architecture, which are server-controlled abstractions made available by an application process and identified by URIs. These server controlled resources are accessed by clients in a synchronous request/response fashion using methods such as GET, PUT, POST, and DELETE of HTTP. Resource state is kept only by the server, which allows for the caching, proxy, and redirection of requests and responses. Web resources may contain links to other resources, which create a distributed web between Internet endpoints, resulting in a highly scalable and flexible architecture. These core web concepts are commonly described as Representational State Transfer (REST) [20]. Today, HTTP is almost exclusively used on today's Internet for manipulating web resources, although REST can also be realized with other application protocols.

The web is usually used by humans to access hypertext (HTML) and other media via web browsers; however, more often the web is used for communications between machines. Interoperable communications between machines on the Internet is often achieved using web services. There are two general ways of realizing web services: applying REST for the manipulation of resources using HTTP, or via Remote Procedure Call (RPC) style interactions using, say, the Simple Object Access Protocol (SOAP).

**Figure 10.**
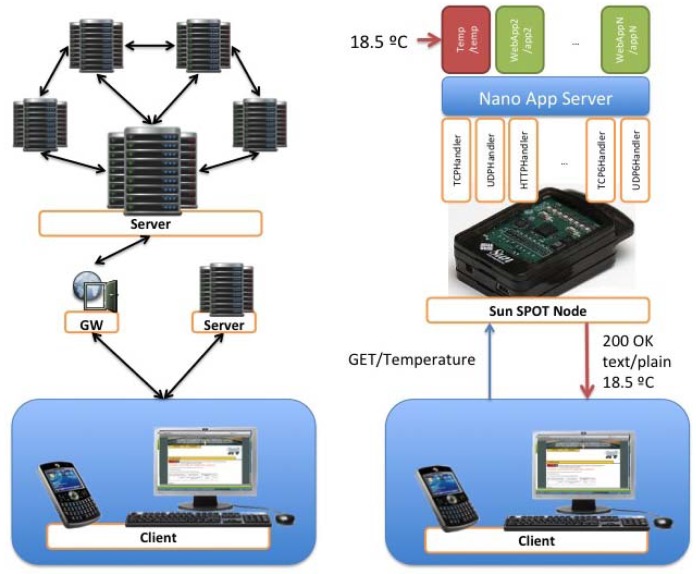
Comparison example between Web Architecture (**left**) and its mapping to a REST Architecture (**right**).

RESTful web services make use of HTTP to access resources, and may be formally described using, for example, the Web Service Description Language (WSDL) or Web Application Description Language (WADL). An HTTP request message is first sent from the client to the server. The client specifies the resource on the server to request in the form of a URI and method (GET, POST, PUT or DELETE). The server responds with a possible response body and a code (Figure 10). HTTP is able to carry an arbitrary payload using any MIME type and encoding. The body of the HTTP web service message is in a format known by the client and server, often XML following a mutually understood Schema. When designing interoperable communications for constrained embedded devices, the RESTful paradigm has many advantages over RPC style interactions. These include less overhead, less parsing complexity, statelessness, and tighter integration with HTTP.

### 4.2. Constrained RESTful Environments (CoRE)

CoRE is a recent working group at IETF providing a framework for resource-oriented applications intended to run on constrained IP networks. A constrained IP network has limited packet sizes, may exhibit a high degree of packet loss, and may have a substantial number of devices that may be powered off at any point in time but periodically “wake up” for brief periods of time. These networks and the nodes within them are characterized by severe limits on throughput, available power, and particularly on the complexity that can be supported with limited code size and limited RAM per node. More generally, we speak of constrained networks whenever at least some of the nodes and networks involved exhibit these characteristics. Low Power Wireless Personal Area Networks (LoWPANs) are an example of this type of network. Constrained networks can occur as part of home and building automation, energy management, and the Internet of Things.

The CoRE working group will define a framework for a limited class of applications: those that deal with the manipulation of simple resources on constrained networks. This includes applications to monitor simple sensors (e.g., temperature sensors, light switches, and power meters), to control actuators (e.g., light switches, heating controllers, and door locks), and to manage devices.

The general architecture consists of nodes on the constrained network, denominated “Devices”, that are responsible for one or more Resources that may represent sensors, actuators, combinations of values or other information. Devices send messages to change and query resources on other Devices. Devices can send notifications about changed resource values to Devices that have subscribed to receive notification about changes. A Device can also publish or be queried about its resources. Typically a single physical host on the network would have just one Device but a host might represent multiple logical Devices. The specific terminology to be used here is to be decided by the working group. As part of the framework for building these applications, the working group will define a Constrained Application Protocol (CoAP) for the manipulation of Resources on a Device [21].

**Figure 11.**
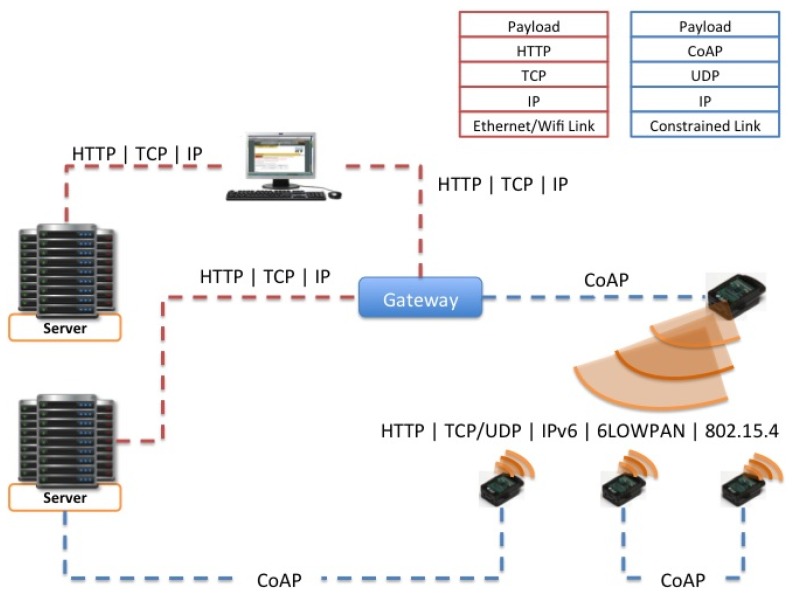
Description of the CoRE communications architecture.

CoAP will be designed for use between Devices on the same constrained network, between Devices and general nodes on the Internet, and between Devices on different constrained networks both joined by an Internet. CoAP targets the type of operating environments defined in the ROLL and 6LOWPAN working groups, which have additional constraints, compared to normal IP networks, but the CoAP protocol will also operate over traditional IP networks. One view of the CoRE architecture is shown in Figure 11.

There also may be proxies that interconnect between other Internet protocols and the Devices using the CoAP protocol. The working group will define a mapping from CoAP to an HTTP REST API; this mapping will not depend on a specific application. It is worth noting that proxy does not have to occur at the boundary between the constrained network and the more general network, but can be deployed at various locations in the unconstrained network.

CoAP will support various forms of “caching”. For example, if a temperature sensor is normally asleep but wakes up every five minutes and sends the current temperature to a proxy that has subscribed, when the proxy receives a request over HTTP for that temperature resource, it can respond with the last seen value instead of trying to query the device that is currently asleep.

Right now, the development of CoAP is being extended with support from ZigBee Alliance, which is extending its current profiles for home automation, consumer devices, healthcare services and smart grid with this new generation of embedded web services. In addition, remark that at the same way that 6LoWPAN does, ZigBee is also considering its extension to the Internet of Things, with its ZigBee-IP stack. On this way it is also being supported by operating systems for Wireless Sensor Networks such as Contiki OS, in addition to the SunSPOT platform proposed in this work, offering between its new features, available in Contiki 2.5, an experimental implementation of the IETF CoRE group's CoAP application layer protocol for RESTful interaction with low-power IP sensor networks.

### 4.3. Nano Application Server

**Figure 12.**
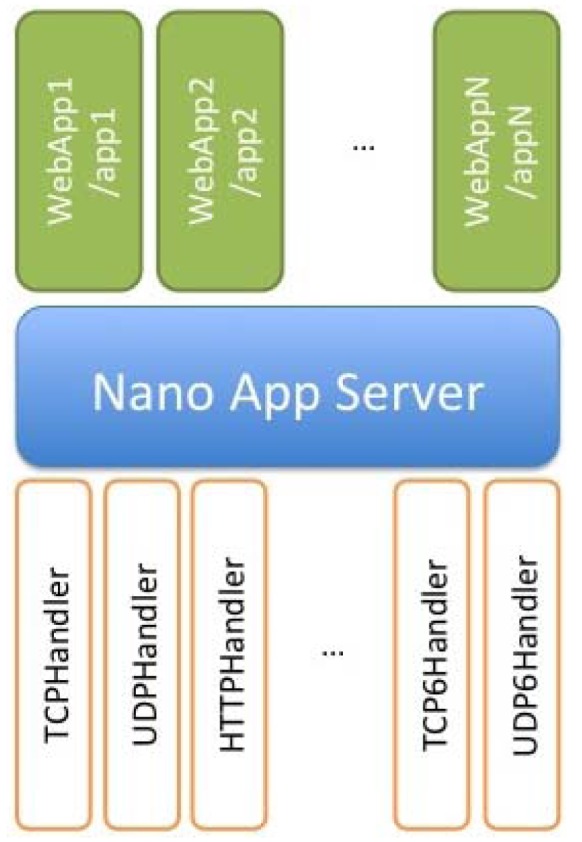
Nano Application Server module architecture.

To carry out the project, it has been necessary to implement an application server running on the different nodes, through which it is possible to access the information provided by each of the applications that run on it. These applications have a similar structure to servlets, and run on a server named Nano Application Server. Web applications run on eSPOT and host nodes and should be registered in the server to manage it correspondent part of the URL subspace. The Nano Application Server supports compressed HTTP and multiple request channels. The Nano Application Server architecture is shown in Figure 12.

The access to the different Web Applications registered on each Nano Application Server, could be easily accessed in some ways, going from a friendly user interface mashed up in a Web page, directly accessible from a Web browser, to a deeper access to the information in a scripting and code-based way using tools like cURL, making the integration of its retrieving of information feasible in query applications for lately manage the information in an automated manner.

**Figure 13.**
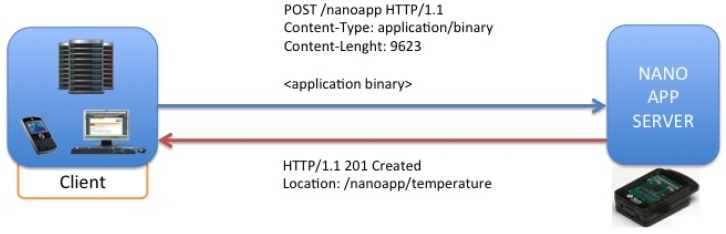
OTA Programming. Creating a resource via REST.

To describe more clearly the behavior of this kind of applications, in the following schemas some examples with the most common applications used in our solution will be shown, among them, the most important are lighting sensor, temperature sensor and RFID inventory retrieval applications. It is possible to do over-the-air (OTA) communication between Sun SPOTs. In order to do that you need to know the IEEE extended MAC address of the SPOTs. The IEEE extended MAC address is a 64-bit address, expressed as four sets of four-digit hexadecimal numbers: *nnnn.nnnn.nnnn.nnnn*. The first eight digits will always be 0014.4 F01. The last eight digits should be printed on a sticker visible through the translucent plastic on the radio antenna fin. A typical sticker would say something like 0000.219 B and that would imply an IEEE address for that SPOT of 0014.4 F01.0000.219 B. In our application, it is easy to address an SPOT due to the automatic discovery process, where the final four digits of the MAC address could identify each SPOT. Applications should not need to create an OTA Command Server explicitly. OTA is enabled or disabled for a SPOT using the ant command line facility. In this way, for deploying or un-deploying applications it needs just to execute the HTTP requests based on REST shown in Figure 13.

Finally, in addition to the automatic discovery process, which is defined for a local domain, it can be used for remote access a human readable and remembered name such as a Uniform Resource Name (URN), or a Network Access Identifier (NAI), e.g., temp_sensor-2C91@lab.um.es, which represents a sensor called temp_sensor-2C91 inside the mentioned domain. Thereby, it is easier to be used for RESTful, where for reaching the temperature value *i.e.*, “temp” property with the next method GET http://lab.um.es/temp_sensor-2C91 temp, it can be referred the node with the http://lab.um.es/temp_sensor-2C91 instead of the IPv6 address in a global level.

**Figure 14.**
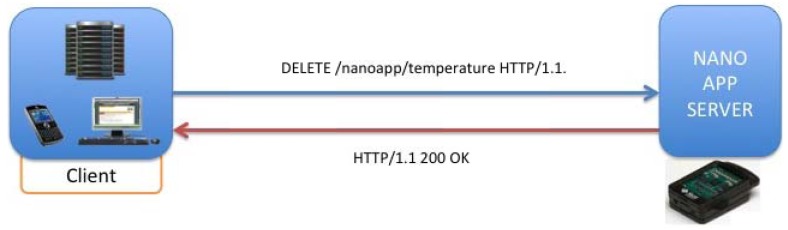
OTA Programming. Deleting a resource via REST.

**Figure 15.**
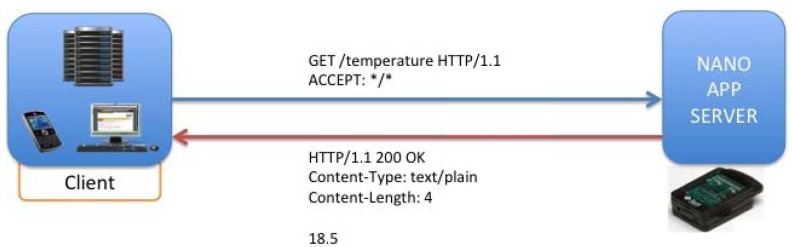
Requesting a resource value.

With the operation described in Figure 14, it is possible to delete resources homed in the SunSPOT nodes, concretely, applications that would be running on the Nano Application Server, being offered as resources addressed by its own URIs. Figure 15 describes the procedure for inquiring a resource value via a GET operation. Concretely it is shown a temperature request over the temperature application hosted in the server.

It is also possible to execute PUT requests, in order to change the behavior of the application, reconfigure it or perform any operation allowed for a concrete resource. PUT operations have been defined for each one of the applications running on the Nano Application Server, in this way, each one has several implemented methods, depending on the purpose of the application, in order to request the information of interest of each application and making possible to perform operations over them when deemed necessary in an straightforward manner.

Related to the OBU services, it is also possible to access them as RESTful resources in order to achieve a great homogeneity level as required by one of the main objectives of the project. For this purpose, the OBU is capable of sending periodically the information related to the GPS coordinates and driver identification credentials that are kept in the TCU centralized system in order to get accessed by the correspondent Web Application registered in the NAS and responsible for this purpose.

### 4.4. Web Access

Applications registered in the NAS for each of the nodes deployed inside the trailer, can be accessed through the Gateway installed on the TCU, which is responsible for managing the requests made from outside in addition to managing the registration of new devices for later access.

**Figure 16.**
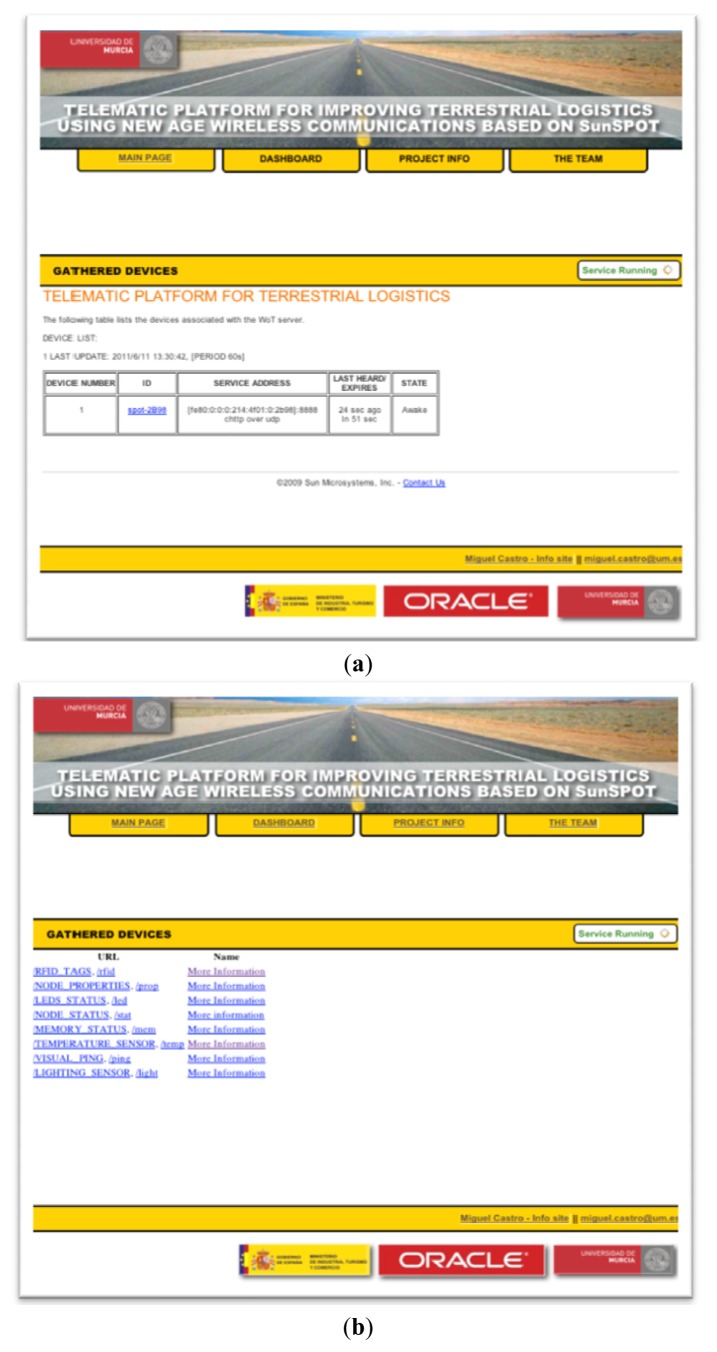
(**a**) Web application for resources discovery; (**b**) Web application for information access.

In order to facilitate access by the client to the various resources offered from the NAS, has been created a Web application (Figure 16), hosted at the Gateway installed on the TCU with an intuitive user interface, through which it is possible access to each other's internal NAS running on the different nodes, being able to consult the states of each of the various applications inside those, through the links provided. Since ultimately these links are references for each of the available resources within each node, when pressed on a particular link, the code snippet is executed for that specific application, hosted on the NAS, responsible for providing the required data, giving its output in plain text so it can be treated later. Moreover it has been considered the possibility of providing the output in a format suitable for JSON (JavaScript Object Notation), HTML or plain text.

**Figure 17.**
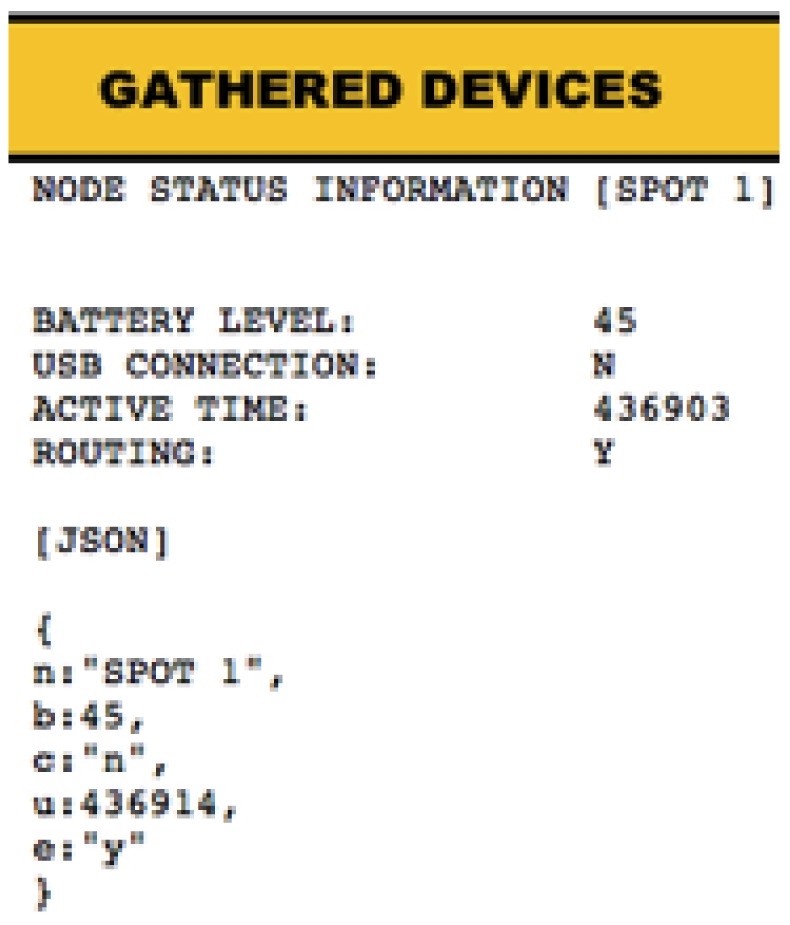
Information output. Plain text and JSON formats.

The use of JSON outputs, allows a back-end information treatment in a higher and more structured (Figure 17) level than in other closed-format outputs. On this way, it is possible to create a versatile set of web applications(e.g., Mash-ups written in AJAX) offering all the available information in the appropriate and required manner depending on the type of user role present at the end of the information retrieval platform.

As described above, with a given output in JSON format, it is possible to access the data in an easy and structured manner. Moreover, using a JSON parser, for example in AJAX, it is offered a straightforward process for treating the information and send it from client to server in a secure manner as if it were an object with its attributes due to the restrictions of the parsing methods, only accepting valid JSON sentences, satisfying then all the security aspects related to that topic. In order to satisfy security aspects, the JavaScript Object Signing and Encryption (JOSE) proposal is also being evaluated for inclusion in future implementations [22].

### 4.5. Block-Wise Transfers

**Figure 18.**
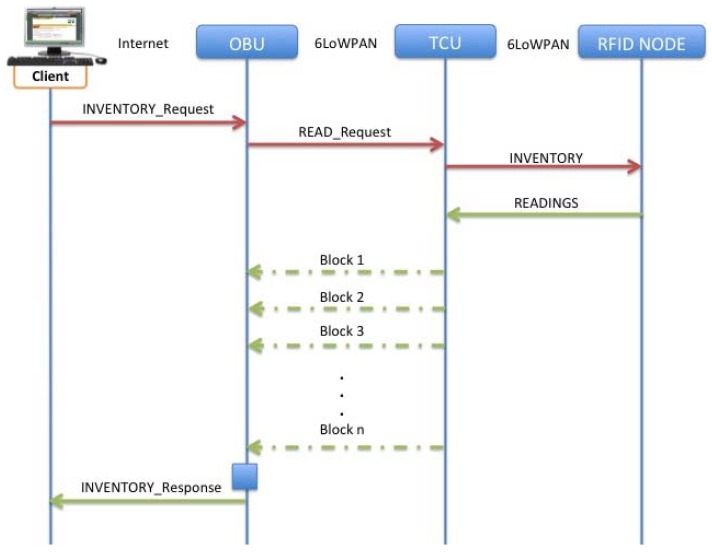
Request and Response interactions based in Block-wise transfers.

Some considerations have been taken into account when designing the solution, in order to satisfy the recommendations explained in the draft of IETF exposed by the CoRE working group, related to block design patterns described in [23]. In this way it is possible to define the request/response between clients and servers (Figure 18) satisfying the commented considerations.

When a resource representation is larger than can be comfortably transferred in a single UDP datagram, a Block option can be used to indicate a block-wise transfer. Using the Block option, a single REST operation can be split into multiple message exchanges, in order to satisfy the requirements on constrained networks such as the ones are involved in the present scenario.

**Figure 19.**
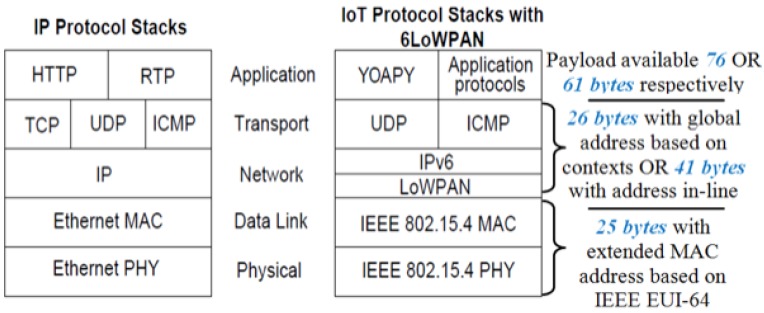
Future Internet of Things Stack (IPv6 & 6LowPAN).

As commented in Section 3.2, there are some reasons for trying to limit the datagram size in low power or constrained networks, in this case, one of the main reasons is the maximum datagram size, approximately 61 to 76 bytes for UDP (see Figure 19) such as other considerations taken in account as the desire of avoiding IP fragmentation and adaptation layer fragmentation in IPv6 and 6LoWPAN respectively [24].

In order to keep the implementation as simple as possible, Block options only support power of two block sizes, in fact, just the range between 16 to 1,024 Bytes is accepted.

#### 4.5.1. Block Options

There exist two different configurations for Block options, named Block1 and Block2 options, both of which are present in request and response messages. However, Block1 is used in request payload operations, while Block2 is used in response payload operations.

Said that, Block1 is useful with payload-bearing POST and PUT requests and their respective responses. On the other hand, Block2 is indicated with GET, POST, and PUT requests and their payload-bearing responses.

Whether the Block1 or Block2 options are used or not, there exist three items of information that may need to be transferred in a Block option: the size of the block (SZX), whether more blocks are following (M), and the relative number of the block within a sequence of blocks with the given size (NUM). Such fields are described in deep in the following lines:

- NUM field: is a variable-size unsigned integer that indicates the block number being requested or provided. Block number zero indicates the first block of a body.
- M field: if unset, indicates that the payload in the message is the last block in the body, if set, it indicates that there are one or more additional blocks available. In case that is being used a Block2 Option in order to retrieve a specific block number, the M bit must be sent as zero and ignored on reception.
- SZX: Block Size. The block size is a three-bit unsigned integer indicating the size of a block to the power of two within the limitations described above.

The block size (SZX) is conveniently encoded as a 3-bit unsigned integer with possible values between 0 for 2ˆ4 to 6 for 2ˆ10 bytes, so the actual block size is then 2ˆ (SZX + 4). This field is transferred in the three least significant bits of the option value. The fourth least significant bit represents the M (More) bit, indicating whether the current block-wise transfer is the last block being transferred, or more blocks are following. Finally, the NUM field is the sequence number of the block currently being transferred, starting from zero.

The default value for both options is zero, indicating that the current block is the first and only block of the transfer, however, there is no explicit size implied by this default value.

It could be useful to implement Block when a REST operation is performed and it is expected that the output will be extremely heavy in terms of data transmission. For example, when an INVENTORY request is done, in order to get all the tags detected inside the trailer, the process of transferring al this information through a constrained network environment could be very overwhelming

It is shown, how satisfying the structure presented in Section 3.2, finally the payload is very constrained in size so applying even more fragmentation could be even worse. Due to that, in order to avoid operations that could cause fragmentation at network level, with the implementation of a block-wise pattern it is possible to carry the fragmentation from network to application layer, being possible to transmit just the explicitly solicited blocks.

With the application of this design patterns it is possible to implement high-level searching techniques such as memory search approaches, leading to future feasible options for optimizing data recovery processes based on Internet of Things.

#### 4.5.2. Conditional Block-Wise

It is considered interesting to introduce a second, and novel, level of fragmentation in order to improve the efficiency of INVENTORY operations such as requesting all the tags read by the RFID reader, but just those of them that fulfills determined precepts. As an example, sometimes it is necessary to check if the amount of products contained inside the truck, belongs to a concrete range of product identification numbers. For this case, it is possible to request all the products whose EPC belongs to a concrete range of product codes in order to obtain certain information about them. This allows getting a specific family of products (e.g., from a milk products shipment only get the list of a specific kind of yogurts).

**Figure 20.**
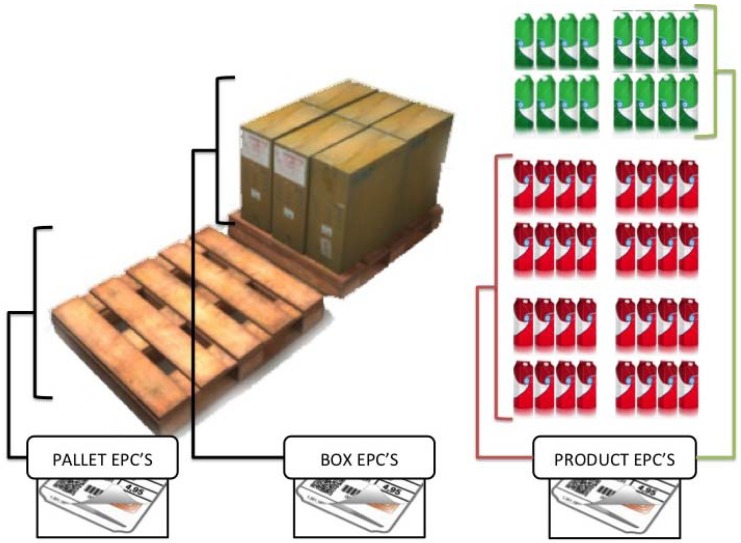
Conditional Block-wise product specification.

This implementation is relevant in scenarios where there could be different types of tags, with different EPC ranges that define different types of packaging methods. In this case, it could be interesting to identify just the pallets inside a truck that are containing an specific type of box/product, or just the boxes inside the cargo area that are containing an specific subset of products (see Figure 20).

### 4.6. Remote Control Server

**Figure 21.**
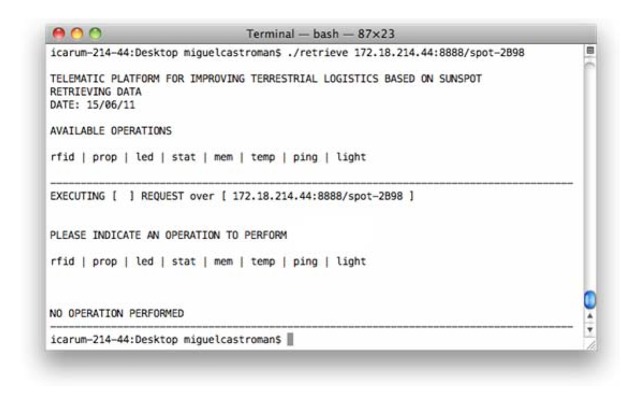
cURL information access example. Available operations.

At the same time as the various resources located in the Nano Application Server running on each node can be accessed through the website developed for this purpose, these resources can also be accessed, consulted and/or modified through the use of platforms such as cURL (Figure 21), which offers the possibility for executing queries in an equally intuitive manner, but also enabling the system to access in a lower level the various resources available in order to support information needs and collection of more detailed data.

Thanks to this, it is possible, for example, to carry out the execution of scripts that will be responsible for collecting some information from the various nodes deployed periodically communicate over the network, to check the status of the remedies in the time intervals determined for each of them. Thus, it is possible to perform various preset data task collection, scheduling activities and making other processes subsequent data analysis, being capable of storing all information concerning a resource to facilitate the task for example, of an audit state of the transport of a product over the distance. It is also possible the installation of alarms and triggers that are activated by the occurrence of a previously established event.

#### 4.6.1. Observing Resources in CoAP

For the development of the proposed solution, some design aspects have been considered in order to have the possibility to support the new design patterns proposed by the IETF in the frame of the CoRE working group, whose aims were described in a previous section. For the concrete case of Remote Control Servers it is interesting to define an approach for the implementation of a Subject/Observer design pattern [25], in the way of subscription protocols.

**Figure 22.**
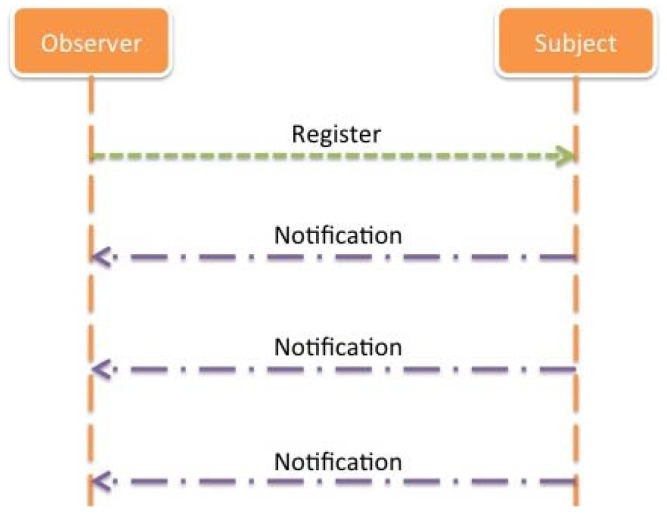
Subject/Observer Design Pattern.

In the subject/observer design pattern, an object, called the subject, maintains a list of interested parties, called observers, and notifies them automatically when a predefined condition, event or state change occurs (Figure 22). The subject provides a way for observers to register themselves with the subject. This pattern supports a clean separation between components, such as data storage and user interface.

**Figure 23.**
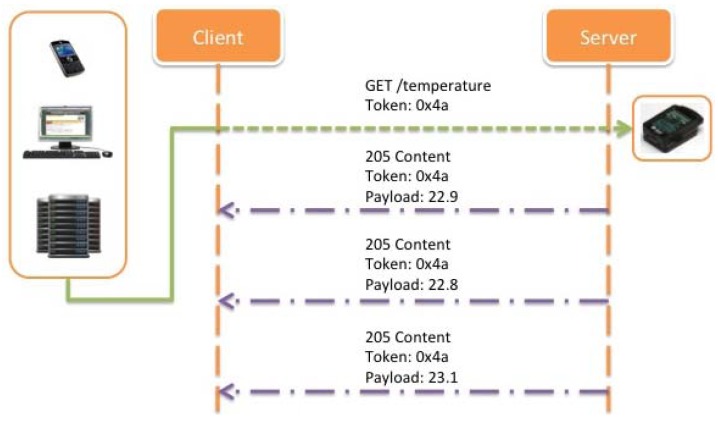
Observing a Resource in CoAP.

Figure 23 shows an example of a CoAP client establishing an observation relationship to a resource on a CoAP server and being notified, once upon registration and then whenever the state of the resource changes.

The design pattern is realized in CoAP as follows:

- **Subject**: In the context of CoAP, the subject is a resource located at some CoAP server. The state of the resource may change over time, ranging from infrequent updates to continuous state transformations.
- **Observer**: The observer is a CoAP client that is interested in the current state of the resource at any given time.
- **Observation Relationship**: A client registers itself with a resource by sending a modified GET request to the server. The request causes the server to establish an observation relationship between the client and the resource. The response to the GET request supplies the client with a representation of the current resource state.
- **Notification**: Whenever the state of a resource changes, the server notifies each client that has an observation relationship to that resource. The notification is an additional response to the GET request; it supplies the client with a representation of the new resource state. The response echoes the token specified in the request, so the client can easily correlate notifications.

Clients could be automatically removed from the list of observers when they are no longer interested in the observed resource. In this case, the server determines the client's interest from the acknowledgments of confirmable notifications, so if a client wants to receive notifications after it has been removed from the observers list, it needs to register again.

The Observe Option modifies an initial GET method, requesting the server to add the sender of the Observe petition to the list of observers for a concrete resource. However, if the server is unable to add the client to the list of observers, it will automatically reply with a simply GET request. Furthermore, the server could adjust this options in order to increase the battery lifetime, so in case that the battery level goes below a determined percentage, it is possible for example to reject every Observe request, replying with a simple GET request instead of registering the petition for being notified every time that the resource change its value or state.

#### 4.6.2. Registration and Notification Procedure

In the registration step, a client can register its interest in a concrete resource offered by the server by performing a GET operation, including an empty Observe Option. In a successful operation the server should return the response, including an Observe Option as well, notifying in this way that the server has subscribed the client to the local list of observers interested in the value or resource requested, so the client will be, since that moment, notified of every change that takes place over this concrete target.

Notifications are additional responses sent by the server replying an initial GET request. For each notification received, an Observe Option with a concrete sequence number, a Token Option, and a payload of the same media type as the initial response will be included. Notifications can be confirmable or non-confirmable, on this way, if a client could not recognize the token in a notification; it must not acknowledge the message, rejecting it with a RST message. Otherwise, an ACK confirmation message will be send.

#### 4.6.3. Conditional Observe Pattern

As seen, when an observe pattern is used CoAP clients can obtain a notification response whenever the observed resource changes. However, sometimes it could be necessary just to get a response when the resource value varies between defined ranges, or a concrete situation happens about which one wants to be notified. This situation could be achieved using a Conditional Observe Option avoiding irrelevant and unnecessary traffic. On this way, Conditional Observe allows implementing an Observe request where notifications will only sent when the established condition occurs [26].

With this new implementation of Observe, the option includes the desired condition for being notified by the server. Thus, when the resource state changes on the server, before sending the notification directly to all its subscribers, it checks the condition previously established, sending the notification only when it is necessary. In order to offer the Conditional Observe, the server needs to follow the general Observe procedure, as described before, but taking into account the special requirements for the Condition option. Since this option is elective, if the server does not support the Condition Option, it will automatically execute a standard Observe procedure. Condition options could be present more than once, meaning that the client has multiple conditions for notification requirements. The following fields compose a Condition option:

- **TYPE field:** defines the condition type. This type is represented by a 4-bit integer, indicating the type of condition being used in the Observe request. Each number of TYPE, represent the type ID of a specific condition type (See Table 3).

Related to the possible values for the TYPE field, and as shown in Table 3, ID. 1 represents the minimum response time. When present, it indicates that the condition required by the client is the minimum response time. The value of this condition is set in the payload of the request, indicating the minimum time the server waits until sending the next notification response. ID. 2 represents the step change value. When present, this value indicates the minimum state change of the value or resource of interest before the server sends a new notification response. Finally, ID. 3, when present, means that the condition is related to a value range. This condition indicates that only when the state value is under the range of this condition, the server will send a new notification response.

- **M field:** indicates the method being used. This field is represented by a 2-bit integer, indicating the method used in the condition as shown in Table 4.
- **VAL field:** condition value. Is a variable-size (4-, 12-, or 20-bit) unsigned integer, indicating the value of the condition.

**Figure 24.**
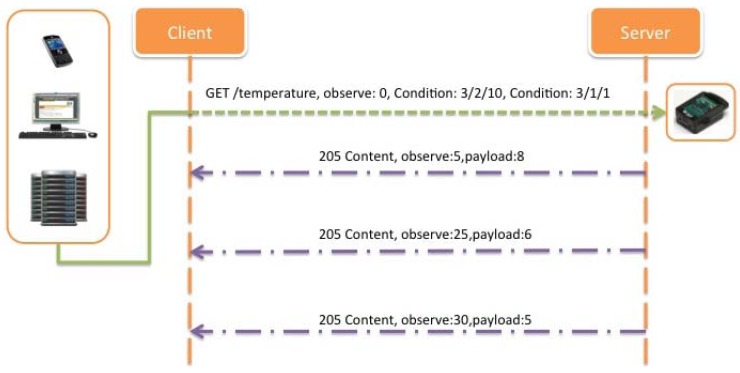
Observing a Resource with conditional operations. Inside Range example.

Note that if multiple conditions with the same condition type are present in the same request, the priority is the same for all of them, and the relationship is “AND”. Figure 24, shows an example of an inside range alarm implementation, where the client adds two different range Condition options, one set to 3/2/10, and the other set to 3/1/1, meaning that the server will only notify when the temperature value monitored is below 10 °C (3/2/10), or above 1 °C (3/1/1).

**Figure 25.**
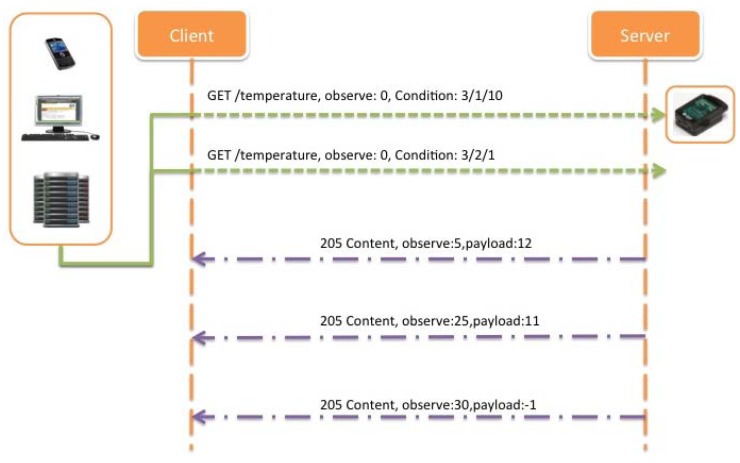
Observing a Resource with conditional operations. Out of Range example.

However, it could be more interesting to receive notifications just when an out of range alarm occurs, instead of sending successive confirmations for a normal operation of the system when the temperature is varying inside the range defined. In this case, and due to the definition of the Condition options, the only way for inquire this type of notifications is using two Condition options, instead of just one in order to define an out of range alarm notification. Figure 25 describes this possibility where the range of interest is the same as described above but notifications are only sent when the temperature values are above 10 °C (3/1/10) or below 1 °C (3/2/1). This is proposed also a novel conditional observe type based on ranges, which is the required for ITS applications.

#### 4.6.4. Example of Operation

In the following lines an example of operation is shown where for a log of temperature values registered during one hour it is possible to see how with different communication patterns it is possible to avoid unnecessary communication processes in order to preserve the battery level of the sensors and avoid network overloads. Note that this section is focused on present a proof of concept, an exhaustive evaluation of each one of the patterns presented and the proposed improvements are carried out in the evaluation section.

**Figure 26.**
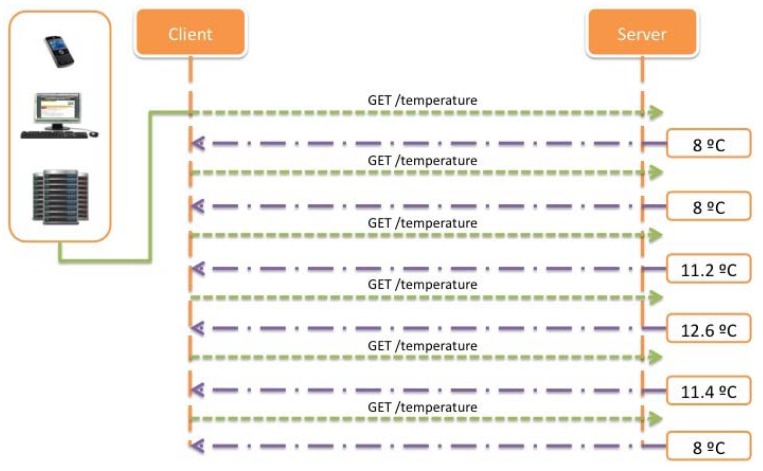
Requesting temperature values with simple polling operations.

Figure 26 shows an example of the registered temperature values for one hour of operation. In the following lines it is shown how implementing different approaches for data retrieving is it possible to reduce the number of necessary transactions. For the most basic case, it is shown how the system would operate with an extremely simple approach based on continuous polling operations, where the client is periodically requesting the value for a resource and waiting for the correspondent reply from the server.

As shown, for the monitoring of the temperatures during one hour, and taking into account that the system will need to check the temperature every 10 minutes, there are needed 12 transactions between the client and the server, *i.e.*, six for requesting the new temperature value and six for providing the response with the associated value.

**Figure 27.**
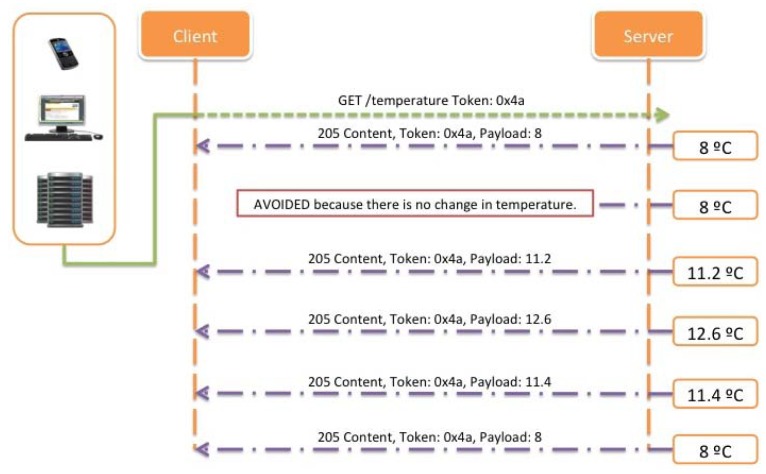
Observe implementation.

Proposing an improvement in order to reduce the number of transactions and keeping in mind that the system should be equally informed about critical situations, it is possible to implement a better method for requesting data from the sensors deployed, in this case, using a straightforward Observe pattern. For the concrete case exposed, the number of transactions will be reduced to six because of the implementation of a registering process where the client is only informed when the value or state of the resource changes (See Figure 27).

**Figure 28.**
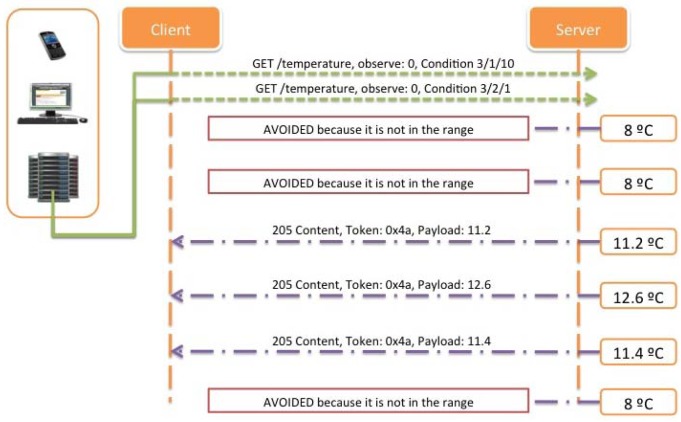
Conditional Observe implementation.

Finally, with the idea of decreasing even more the number of transactions needed, and taking into account the benefits of using an improved version of the Observe pattern, it is shown (Figure 28) how using the Condition Observe it is possible to reduce the load of the network, and at the same time lengthen the battery life of the deployed nodes.

With the implementation of Condition Observe, the number of operations necessary to inform the client has been reduced to five (two for registering the conditions in the server and three replies with the required information). Related to that, it could be possible to reduce even more this number improving the protocol in order to set more powerful conditions such as priority conditions or providing the possibility for using other type of operators such as OR instead of just AND conditional operations. Some contributions in this topic are being studied for future improvements leading to lightweight implementations.

Note that this implementation is not only interesting for the specific case of temperature monitoring, but it is also feasible to use in any other type of sensors or devices connected through a network with the requirements described. An example of this is the possibility to introduce this type of conditional transferences between the system and the integrated RFID reader, in order to reduce the number of transactions taking into account that it is possible to condition read operations performed by the RFID reader when it is necessary, discriminating certain type of tags based on different aspects.

## 5. Evaluation

**Figure 29.**
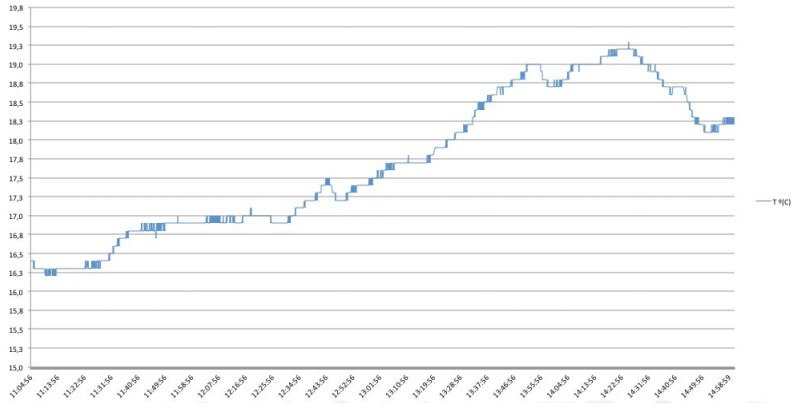
Temperature measurements during 4 hours.

In order to validate the protocols exposed, and with the aim of demonstrating the ability for reducing the number of necessary messages sent through the network, one temperature dataset obtained from the United States Geographical Service (USGS) [27], related to the temperature in Celsius degrees in Stillaguamish River Basin, Washington, August 2011 has been taken as an example, where temperature measurements were done each 3 seconds during an interval of four hours approximately. Figure 29 shows the graph obtained from the retrieved data and in Table 5 the results of implementing different communication patterns are shown.

**Figure 30.**
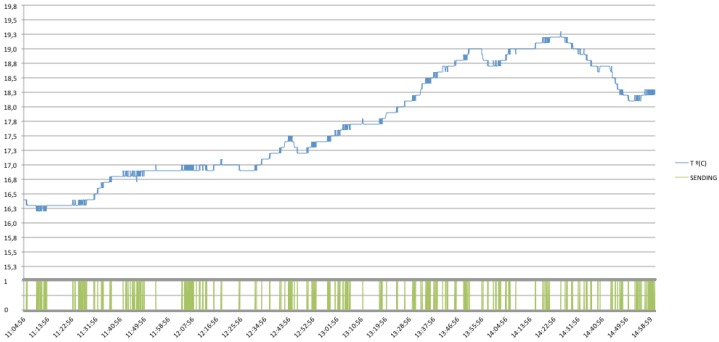
Temperature measurements notifications with Observe pattern.

When operating with the simplest approach, based on continuous requests to the sensor, the number of request messages raises to 4,718 with a total amount of 9,436 messages between client and server (*i.e.*, 4,718 requests and their respective replies). In addition, the sensor is continuously sending and receiving data so the battery levels would decrease very fast, reducing the life-time of the sensor due to an ineffective mode of operation. However, in case of using an Observe pattern, the number of necessary requests descends drastically because there is needed only one subscribing operation in order to be informed for future values, keeping in mind that data, will only be send in case that the sensor detects a change in the measured resource. In this this case, there are needed just 612 messages sent by the server in order to notify the client and satisfy its subscribing requirements, meaning a reduction of the network usage (See Figure 30).

Furthermore, in the following figures the performance of the system will be shown with different communication protocol implementations, concretely, there are shown the different behaviors when different Conditional Observe options are applied (*i.e.*, Minimum Response Time, Step and Range Options). Note that, a Condition Option, is present together with the standard observe request, representing the condition required by the client. Then, the server needs to follow the general procedure as described for the Observe pattern, but taking into account the Condition Option desired.

**Figure 31.**
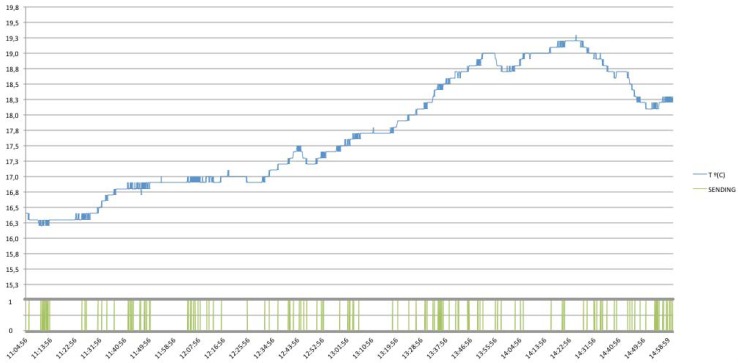
Notifications with Observe Conditional, Minimum Response Time option set to 15 seconds.

In the case of using a Conditional Observe with a Minimum Response Time Option, the number of messages could be reduced or increased proportionally to the time established for sending a notification. Figure 31, shows an example of this implementation, with a Minimum Response Time of 15 seconds, instead of the default sensor configuration, established in 3 seconds.

As shown, it is noticed that the number of necessary messages sent by the server decreases substantially, for the Minimum Response Time established, the total amount of messages sent is 137, and just one from client to server, in order to register its notification conditions.

**Figure 32.**
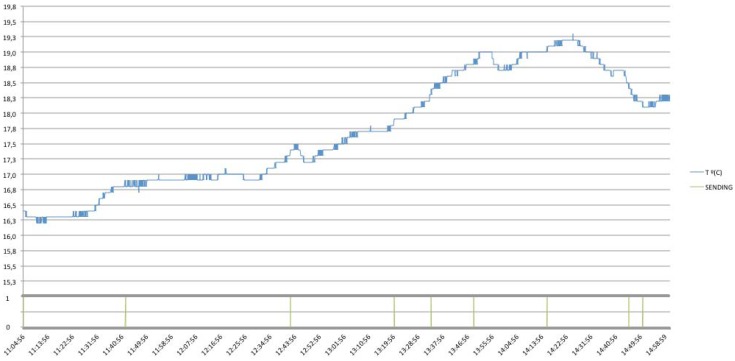
Notifications with Observe Conditional, Step option set to 0.5 °C.

Regarding to the Step Option, we can define the minimum state change of the desired resource before the server needs to send a notification response. In this case, it has been established a Step Option to 0.5 °C. Figure 32, shows the number of messages sent by the server for this concrete case, having a total amount of nine notifications, with just one necessary request by the client, with the desired options.

**Figure 33.**
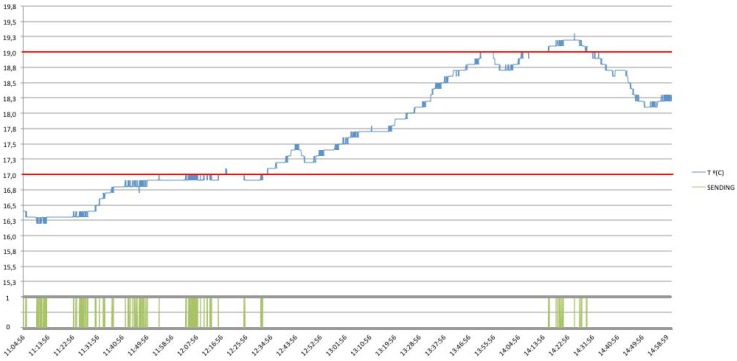
Notifications with Observe Conditional. Range Option (<17.0 °C & >19.0 °C).

Finally, it is shown how thanks to the Conditional Observe, it is possible to reduce even more the number of messages needed, in order to be informed just in case of trespassing critical ranges, reducing the total amount of necessary transactions to the number of 222 (See Figure 33). In this case, two different conditions have been established, in order to be notified when the temperature values are above 19.0 °C or below 17.0 °C.

For the same dataset, it is clear how implementing different methods it is possible to reduce the load of the network about an 87% using an Observe pattern, in comparison with an standard continuous request approach, and finally, with the addition of conditional operations it is possible to reduce even more the number of necessary messages, always depending of the concrete configuration and notification requirements.

Table 5 shows the behavior of the different protocols summarizing the different approaches discussed when applied to the initial temperature dataset. Considering the results, it is shown how it is possible to adjust the protocol in the more appropriated way in order to be sufficiently informed in different situations, such as a concrete time interval (*i.e.*, Minimum Response Time option), when the value change is greater than a determined step (*i.e.*, Step option), or about desired or critical situations within a range (*i.e.*, Range option), instead of being notified at every value change or irrelevant situations in a continuous manner.

## 6. Conclusions

This paper has presented how, involving several technologies from ICT, in conjunction with electronic devices already integrated in the ITS solutions such as GPS/GNSS and GPRS, it is possible to meet the requirements and extend the possibilities of the new-age logistics facilities, offering a wide and versatile enough range of applications and services in order to ensure the quality of the products shipped, making the management tasks and transportation process easier and more efficient.

This project considers the ITS platform defined under the previous works carried out in the frame of the Spanish project SATELITES, where it was built the On-Board Unit of the architecture. Under this work has been designed the Trailer Control Unit, together with the integration of SunSPOT technology.

This work has presented the integration of the different sensors and RFID readers in SunSPOT nodes. The system presented fulfills the objectives for reaching a comprehensive and flexible solution that eases integration and deployment processes, in order to make them scalable. Moreover, this offers high homogeneity level entities, facilitating system configuration tasks in environments with high heterogeneity features through IPv6 connectivity and the RESTful embedded Web Services. Thereby, access to traceability, tracking and monitoring services is available anytime during the course of the transportation of goods. With the purpose of achieving even more flexibility, several ways for accessing the information in real time with a series of Web Services RESTful and information accessing methods such as cURL through an implementation of a Nano Application Server running directly on the monitoring nodes allowing consequently both, H2M (Human to Machine) interaction as well as M2M (Machine to Machine) interaction features has been defined.

SATELITES and related works defined a solution focused on environmental monitoring and product identification. Going one step further, with this approach, based on Internet of Things, is it possible to reach an item-level monitoring, when, due to the critical characteristics of concrete products it could be necessary. For this reason there has been considered some design aspects in order to fulfill this new scalability requirements when necessary. An example of that design aspects are the *Block-wise, Conditional Block-wise* and *Observe* communication protocols with its different configuration options for the concrete case of *Conditional Observe* implementations. On this way, novel implementations based on this pattern have been presented and proposed in order to improve communication aspects and adapting the system to the requirements needed in constrained networks, leading to lightweight communication protocols. Moreover, it has been shown that following the guidelines of network usage improvement in terms of efficiency, and network load reduction, it is possible to optimize resources in order to establish and maintain communication processes in a lightweight manner.

The proposed platform based on Internet of Things allows for a new set of different applications that could be defined, taking advantage of the incremental device intelligence and its communication possibilities leading to a full-duplex communication between the monitoring entities and the goods itself without having to rely on other entities, increasing autonomy, and increasing scalability, which is highly required having in consideration the fact that the Future Internet will be composed by every-nature embedded devices with network access, bridging the everyday smaller gap between real and virtual worlds.

However, this process leads into multiple technical issues and requirements that need to meet, especially when constrained environments take part in this. For this reason, it is necessary to reach unified architectural solutions to make every resource accessible in a straightforward and standard manner, obtaining a scalable continuous monitoring system with the use of the new technologies from the Internet of Things. Next steps in our research work pass through an exhaustive analytical and empirical evaluation in order to obtain a reliable performance evaluation process for measuring the different aspects involved in the area of continuous monitoring, tracking and traceability of goods, and the evaluation of the presented patterns over new protocols such as GLoWBAL IPv6 [28], which are defining a new generation for the communications based on IPv6 for Wireless Sensor Networks.

## Figures and Tables

**Table 1.** Codes of Transponder Types.

**Value**	**Transponder Type**
0 × 80	ISO 18000-6 A
0 × 81	ISO 18000-6 B (UCODE; UCODE EPC 1.19)
0 × 83	EM4222, EM4444
0 × 84	EPC class 1 Gen 2
0 × 88	EPC class 0/0+ Gen 1
0 × 89	EPC class 1 Gen 1

**Table 2.** Codes of Identifier Data Types (IDDT).

**Value**	**IDDT**
0 × 00	SNR or UID
0 × 01	EPC

**Table 3.** Observe Condition types and its identifiers.

**Condition Type**	**ID.**
Minimum response time	1
Step	2
Range	3

**Table 4.** Conditional Observe Methods and its identifiers.

**Method**	**ID.**
Equal to (=)	0
More than (>)	1
Less than (<)	2

**Table 5.** Comparison between the different methods used for data request.

**Method**	**Client Requests**	**Server Replies**	**Network Usage Reduction (%)**	**Function of**
Pooling	4,718	4,718	___	Number of Requests
Observe	1	612	87.03%	Resource value change
**Conditional Observe Options**				
Minimum Response Time (15′)	1	137	97.10%	Resource value change and Minimum Response Time Option established
Step (0.5 °C)	1	9	99.81%	Resource value change and Step Option established
Range (<17 °C & >19 °C)	2	222	95.29%	Resource value change and defined Range Option established
